# In Vivo Distribution of Poly(ethylene glycol) Functionalized Iron Oxide Nanoclusters: An Ultrastructural Study

**DOI:** 10.3390/nano11092184

**Published:** 2021-08-25

**Authors:** Maria Suciu, Claudiu Mirescu, Izabell Crăciunescu, Sergiu Gabriel Macavei, Cristian Leoștean, Rǎzvan Ştefan, Loredana E. Olar, Septimiu-Cassian Tripon, Alexandra Ciorîță, Lucian Barbu-Tudoran

**Affiliations:** 1 Electron Microscopy Centre, Faculty of Biology and Geology, Babeș-Bolyai University, 44 Republicii St., 400015 Cluj-Napoca, Romania; suciu.maria@ubbcluj.ro (M.S.); claudiu.mirescu@gmail.com (C.M.); septimiu.tripon@ubbcluj.ro (S.-C.T.); 2 Integrated Electron Microscopy Laboratory, National Institute for Research and Development of Isotopic and Molecular Technologies, 67-103 Donat St., 400293 Cluj-Napoca, Romania; 3 Physics of Nanostructured Systems Department, National Institute for Research and Development of Isotopic and Molecular Technologies, 67-103 Donat, 400293 Cluj-Napoca, Romania; izabell.craciunescu@itim-cj.ro (I.C.); sergiu.macavei@itim-cj.ro (S.G.M.); cristian.leostean@itim-cj.ro (C.L.); 4 Research Centre for Biophysics, Life Sciences Institute, Faculty of Veterinary Medicine, University of Agricultural Sciences and Veterinary Medicine Cluj-Napoca, 3-5 Manastur St., 400372 Cluj-Napoca, Romania; rstefan@usamvcluj.ro (R.Ş.); loredana.olar@usamvcluj.ro (L.E.O.)

**Keywords:** magnetic nanoparticles, SPIONs-PEG, in vivo tracking, electron microscopy, liver, lung

## Abstract

The in vivo distribution of 50 nm clusters of polyethylene glycol-conjugated superparamagnetic iron oxide nanoparticles (SPIONs-PEG) was conducted in this study. SPIONs-PEG were synthesized de novo, and their structure and paramagnetic behaviors were analyzed by specific methods (TEM, DLS, XRD, VSM). Wistar rats were treated with 10 mg Fe/kg body weight SPIONs-PEG and their organs and blood were examined at two intervals for short-term (15, 30, 60, 180 min) and long-term (6, 12, 24 h) exposure evaluation. Most exposed organs were investigated through light and transmission electron microscopy, and blood and urine samples were examined through fluorescence spectrophotometry. SPIONs-PEG clusters entered the bloodstream after intraperitoneal and intravenous administrations and ended up in the urine, with the highest clearance at 12 h. The skin and spleen were within normal histological parameters, while the liver, kidney, brain, and lungs showed signs of transient local anoxia or other transient pathological affections. This study shows that once internalized, the synthesized SPIONs-PEG disperse well through the bloodstream with minor to nil induced tissue damage, are biocompatible, have good clearance, and are suited for biomedical applications.

## 1. Introduction

Designing a new drug delivery system always brings a series of limitations, regardless of its use and especially when nanotechnology is involved. The ever-growing interest in this interdisciplinary field has attracted numerous funds in the research of the topic [1,2], and, more importantly, extraordinary results have been obtained [3]. The amount of resources invested in superparamagnetic iron oxide nanoparticles (SPIONs) only is concrete proof that no matter how much information is acquired along the way, there are still some questions left unanswered [4]. Such questions are connected to the extent of damages that nanoparticles (NPs) could induce at subcellular levels [5]; the in vivo behavior after in vitro testing [6,7]; and, most importantly, the pathway through which NPs, especially SPIONs, enter the cells [8], after which comes the long term effects that may or may not affect future generations [9,10].

SPIONs are one of the most versatile types of magnetic NPs used in domains such as microbiology [11,12], MRI (magnetic resonance imaging) [13,14,15], and alternative treatment of malignant tumors [16,17,18]. Some of the parameters that decide the fate of SPIONs are related to the synthesis method [6,19], the biocompatible polymers used to form their shell [20,21], and their ‘final destination’. They can be designed to enhance contrast in MRI patients, where they are retained for short periods, or target specific zones in the human body, requiring long-term treatments. However, even if they are considered safe to use with patients [22], the same SPIONs are reported to have toxic effects when tested in different circumstances, either in vitro or in vivo [23]. Metal nanoclusters are a new type of platform that have different characteristics than singular nanoparticles. (Ultra)small nanoclusters (<20 nm) have been proven to have increased efficiency for catalytic, photoluminescence, coulorimetric, chemodynamic, and sensor activity in comparison with large-size nanoparticles [24,25,26,27,28]. However, due to their size, they can interfere with normal metabolic activities in the cells.

Many important breakthroughs have been achieved with the use of magnetic NPs. Cancer treatment through chemotherapy and radiotherapy is still chosen over alternative therapies involving nanotechnology. On the other hand, the side effects of conventional cancer treatments are undoubtable, and development of a method for targeted treatment of cancer using SPIONs is ongoing in clinical trials [29]. SPIONs can be ideal carriers for chemotherapeutic drugs [30], therefore reducing the overall toxicity on the patient by targeting only the affected zone [3]. Deh et al. (2020) [16] achieved a successful reduction of tumors in mice after SPION administration and hyperthermia treatment [16]. Butoescu et al. [31] achieved a controlled drug release in the joint cavity of mice with the help of SPIONs as an alternative treatment of arthritis and osteoarthritis. SPIONs loaded with a drug (dexamethasone) were kept in place using an external magnetic field to maintain a relative constant drug release in the hope of reducing damages in surrounding tissues [31].

An in vitro simulation of the blood–brain barrier demonstrated that SPIONs could inhibit neurotoxicity, and become a potential solution for future treatment of brain tumors [32]. Another successful use of SPION hyperthermia was reported by Kandasamy et al. [33] in their work. After in vitro treatment of hepatic cancer cells with SPIONs, no morphological changes were observed, but when hyperthermia was induced, an increase in cell death was registered, further indicating that unless directed to a specific region and magnetically activated, SPIONs have no harmful effects on the healthy tissues [33].

However, another study revealed that a significantly high dose of SPIONs is necessary to achieve proper tumor inhibition through hyperthermia [34]. The studies presented above used concentrations ranging from 5 µg/mL to 20 mg/mL SPIONs. This indicates that for the hyperthermia achieved in vitro using fewer SPIONs, a higher concentration of Fe is required for in vivo reduction of tumors. However, SPIONs may accumulate inside organs such as the liver and kidneys [15], causing organ impairment or failure [35].

It is essential to address the issues concerning in vivo distribution and clearance of SPIONs. After successful synthesis of SPION-PEG (polyethylene glycol) clusters and administration in normal human keratinocytes and skin melanoma cells in previous work [8], their effects in vivo on Wistar rats were further assessed. Following short-term and long-term exposure to SPIONs-PEG, the liver, kidney, lung, skin, brain, and spleen were investigated histologically and at ultrastructural levels. Blood and urine samples were collected during treatment and were examined through fluorescence spectrophotometry, confirming that SPIONs-PEG entered the system.

Regardless of the time that passed after injection, the designed SPION-PEG cluster administration had minimum side effects and sporadically accumulated in several organs, indicating that they spread throughout tissues (not just filtering organs). It was often shown that only small singular SPIONs (<30 nm) could cross biological barriers and that the reticulo-endothelial system rapidly captured larger SPIONs (>100 nm) and so they were removed from circulation before reaching the targeted organs [36,37]. Our 50 nm SPION-PEG clusters were able to cross the endothelial barriers and reach the kidney, which has a filter slit of approximately 5.5–10 nm [38,39]. These aspects were better noticed using transmission electron microscopy (TEM), as the small, isolated nanoparticles can be identified easily in the tissue’s background. In this regard, the SPION-PEG clusters designed here may offer high applicability for targeted tumor treatment or other biomedical applications, since a higher concentration of Fe within a small dose per administration could facilitate hyperthermia.

## 2. Materials and Methods

### 2.1. SPION-PEG Clusters Synthesis and Characterisation

SPION-PEG clusters of 50 nm were synthesized and characterized as previously described [8]. First, 10 nm Fe_3_O_4_ crystallites were obtained through a coprecipitation method under hot conditions, then cooled precipitates were magnetically separated from the remaining reactants, washed, and dried. The obtained particles were coated and packed in 50 nm spherical clusters using an oil-in-water emulsion method, separated, and then dispersed in water [40,41].

To determine the crystalline structure and morphology and to confirm the superparamagnetic nature of SPIONs-PEG, TEM and X-ray powder diffraction (XRD) (analyses were performed. At the same time, the magnetic behavior was characterized through a vibrating sample magnetometer (VSM). For TEM, the water-dispersed sample was placed on a 200 mesh copper grid and imaged using a scanning-transmission electron microscope (STEM) Hitachi HD2700 (Hitachi, Tokyo, Japan) at 200 kV, and coupled with EDX (energy-dispersive X-ray) detector (Oxford Instruments, Oxford, UK, AZtec Software, version 3.3) used for elemental detection. Dynamic light scattering (DLS) analysis, zeta potential, and the polydispersity index were measured by dispersing the SPIONs-PEG clusters in phosphate buffer saline at concentrations from 0.1–500 μg/mL and read on a Zetasizer Nano ZS90 (Wyatt Technology Corporation, CA, USA) at 25 °C, pH 7.4, 90° reading angle. XRD analysis was done using a high-resolution SmartLab X-ray diffractometer (Rigaku, Tokyo, Japan) operated at 9 kW and coupled with SmartLab Guidance software (SmartLab Studio II package software, Rigaku, Tokyo, Japan). The magnetic behavior of the samples was recorded using a vibrating sample magnetometer (VSM) produced by ‘Cryogenic Ltd.’ (London, UK), at room temperature and magnetic fields up to 5T.

Before in vivo administration, the absorbance spectrum of SPIONs-PEG was recorded with the UV-VIS spectrometer PerkinElmer Precisely Lambda 25 (PerkinElmer Inc., Waltham, MA, USA) at room temperature, in the range of 200–800 nm and with a resolution of 2 nm.

### 2.2. In Vivo Distribution and Clearance Analyses

#### 2.2.1. Animal Model and SPION-PEG Clusters Administration

All animal procedures were in accordance with the institutional guidelines and ethical standards approved by the ‘Babes Bolyai’ University of Cluj-Napoca, Romania. SPIONs-PEG were administered through intraperitoneal (i.p.) or intravenous (i.v. in the caudal vein) injection to male Wistar rats (*N* = 9) of 300–450 g weight (1 mL saline containing the equivalent of 10 mg Fe/kg body weight). Each animal’s behavior was monitored for 15 min after the injection to prevent any painful reaction development. At the below-mentioned time points, rats were anesthetized with ether vapors, and their backbone dislocated, and the following procedures were done after no response was received from pressure applied to the members [42]. The organs and body fluids were collected at 15, 30, 60, and 180 min and 6, 12, and 24 h for i.p. administration, and 24 h for i.v. administration. The time intervals were chosen for short- and long-term investigations. One rat received physiological serum (saline) i.p. with as a control model.

#### 2.2.2. Identification and Distribution of SPIONs-PEG in Blood and Urine by Fluorescence Spectroscopy

The spectrofluorimetric analysis of blood and urine samples was performed at room temperature using a Jasco Fp 8200 spectrofluorometer (JASCO International Co., Ltd., Tokyo, Japan). For the increased resolution of detection of the spectral changes associated with SPIONs-PEG, blood samples were diluted 500-fold with phosphate-buffered saline (PBS). In contrast, the urine samples (2 mL) did not undergo any processing or centrifugation before analysis. No urine was available at 15, 30, and 60 min post-administration, these results are therefore not shown. Fluorescence of serum and urine samples was measured at 410 nm (for serum) and 320 nm (for urine). The signal intensity was measured at the maximum emission of 480 nm and 513 nm, respectively. The fluorescence intensity of the urine samples was measured at an emission wavelength of 420 nm. The fluorescence data were analyzed using OriginPro 8.5.1 software (OriginLab Corporation, Northampton, MA, USA).

#### 2.2.3. In-Tissue Identification and Distribution of SPION-PEG Clusters and Histopathological Analyses Using Light and Electron Microscopy

For SPION-PEG cluster distribution and biocompatibility analysis, we performed hematoxylin-eosin staining on several organs, including liver, spleen, lung, kidney, brain, and skin. The collected organs were prepared for histopathological examination according to Mirescu et al. [43]. After fixation with 4% paraformaldehyde, the samples were washed in PBS, dehydrated with xylene, and included in melted paraffin. Then, 5 µm thick sections were obtained using an automated microtome mounted on glass slides, deparaffinized at 50 °C, and rehydrated. The sections were stained with hematoxylin for 4–10 min depending on the tissue, and eosin for 2 min, then permanently fixed with Canadian balm. A trained histopathology-specialized medical doctor analyzed the sections.

For TEM analyses, the organs were prepared using an adjusted protocol after Hayat [44] and according to Craciun and Barbu-Tudoran [45]. Immediately after harvest, 1 mm^3^ tissue samples were fixed with 2.7% glutaraldehyde in 0.1 M PBS for 1.5 h and then with OsO_4_ (2% in 0.15 PBS at pH 7.4) for 1 h. Next, the samples were dehydrated, included in epoxy resin (Epon 812), and cured until hard.

Semi-thin sections (250 nm thickness) were obtained using an ultramicrotome (Leica UC7) and were stained with epoxy tissue stain for supplementary histological analysis. Images were taken using the light microscope Olympus BX51 (Olympus, Hamburg, Germany), coupled with CCD camera CoolSNAP-Pro (Olympus, Hamburg, Germany).

The ultrathin sections (70–90 nm) were collected on 200 mesh copper grids, double-stained with uranyl acetate (2.6 g in 20 mL of 50% ethanol in ultrapure water, for 6 min) and lead citrate (1.41 g in 42 mL MQ water and 8 mL NaOH 1 N, pH 12, for 3 min). Samples were examined using a TEM Jeol JEM 1010, and electron micrographs were obtained with MegaView III camera (JEOL, Tokyo, Japan). To obtain relevant qualitative analysis results, at least 3 pieces of tissue sample were collected from different parts of the selected organs. From each sample, three grids were obtained from different places within the sample thickness.

To identify intracellularly dispersed Fe_3_O_4_ deposits, EDX analyses were used on tissue samples. Analyses were done on a scanning electron microscope (SEM) Hitachi SU8230 (Hitachi, Tokyo, Japan) at 30 kV coupled with Oxford Instruments EDX detector and the elemental composition was obtained in AZtec Software (Oxford Instruments, Oxford, UK, AZtec Software, version 3.3).

## 3. Results

### 3.1. SPION-PEG Clusters Characteristics

**Figure 1:**
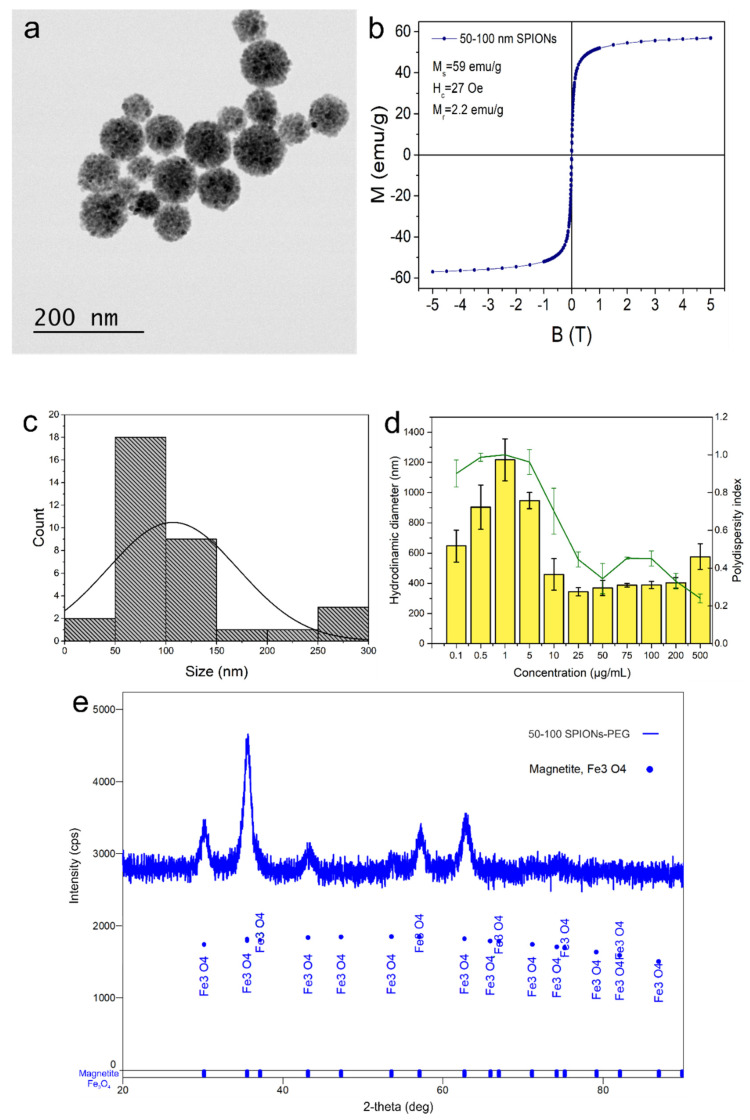
TEM micrograph of the obtained SPION-PEG clusters (**a**), with the magnetization curves versus applied magnetic field at room temperature (**b**), their size distribution (**c**), and hydrodynamic diameter as determined through dynamic light scattering (**d**). XRD pattern of the resulted SPION-PEG clusters and compared to P.D.F. card 01-076-7165 of magnetite (**e**).

TEM analysis of SPIONs-PEG revealed relatively uniform clusters with a median diameter of 50 nm (narrow distribution between 50 and 80 nm). Clusters contained ~10 nm individual SPIONs, held together by a visible layer of PEG (Figure 1a). DLS measurements of SPION-PEG clusters (Figure 1d) revealed a hydrodynamic dimension ranging from 300–1200 nm (in phosphate buffer). At 1 µg/mL concentration, it registered the highest dynamic size (1200 nm) and the worst polydispersity index (~1). Still, at higher concentrations (10–500 µg/mL), the clusters seemed to have better dispersity indexes (0.2–0.4) and lower hydrodynamic diameters (approx. 300 nm). The measured zeta potential was −19 ± 0.4 mV.

The room temperature magnetization curves of SPIONs-PEG are shown in Figure 1b. The shape of the magnetization curve was characteristic of superparamagnetic samples with interacting particles, displaying a very small hysteresis loop. The saturation magnetization was M_s_ = 59 emu/g, the coercive field was H_c_ = 27 Oe, and the remnant magnetization was M_r_ = 2.2 emu/g.

The XRD analysis confirmed that the samples were magnetite (Fe_3_O_4_) (Figure 1e). Five distinctive diffraction peaks at 2θ values (30.26°, 35.63°, 43.17°, 57.19°, and 62.68°) that corresponded to the reflection planes of (220), (311), (400), (333), and (440) characteristic to the cubic-centered face of magnetite were observed. The crystalline size, as determined through the Williamson–Hall method, was of 50(12) Å, indicating the successful formation of magnetite.

### 3.2. SPION-PEG Cluster Distribution and Clearance

In this study, we focused on the bio-distribution, histopathology, and ultrastructural effects of SPION-PEG clusters administered by i.p. injection. Due to the existence of a plethora of studies analyzing SPION distribution after i.v. administration, relatively fewer bio-distribution studies for i.p.-administered SPIONs [46], a limited number of animals for testing (for ethical reasons), the possibility of a better outcome for the rats, improved pharmacokinetic profile, and bioavailability for large kDa drugs after i.p. administration [47,48,49,50], we chose to test for SPION-PEG cluster in vivo effects in this administration setup. Organs (liver, spleen, lung, kidney, brain, and skin) were examined at different time intervals: 15, 30, 60, and 180 min, and 6, 12, and 24 h after i.p. administration, respectively. Based on the observed results, SPION-PEG clusters were also intravenously administered, and the same organs and body fluids were examined at 24 h only.

### 3.3. Blood Serum and Urine Spectrofluorimetric Studies

The in vivo effects of SPIONs-PEG clusters were investigated at two different time intervals to better understand the nanoparticles’ distribution through filtering organs. Blood and urine samples were analyzed through fluorescence spectrophotometry. Prior to administration and spectrofluorimetric investigation of the biological fluids, the optical properties of SPION-PEG clusters were investigated by UV-VIS. The UV-VIS spectrum revealed three absorbance peaks at ~320 nm, ~410 nm, and ~540 nm.

#### 3.3.1. Blood Serum—i.p.

**Figure 2:**
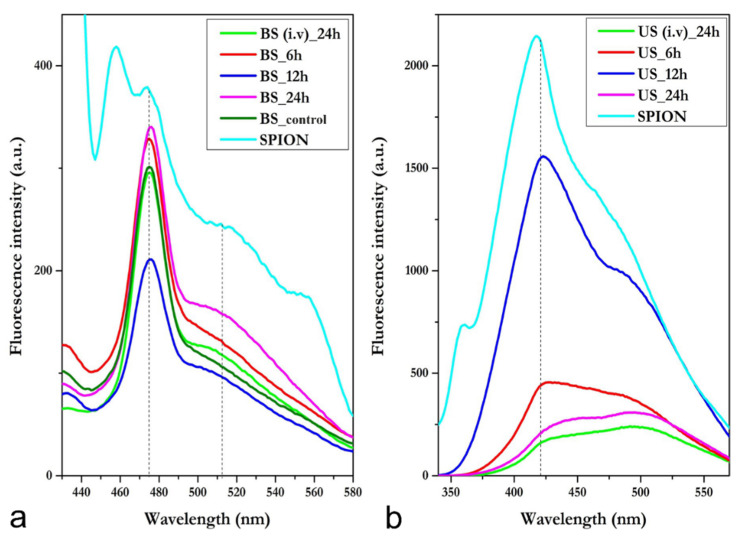
The fluorescence spectra of blood (**a**) and urine (**b**) samples collected at 6, 12, and 24 h post-intraperitoneal administration and 24 h post-intravenous administration of SPION-PEG clusters. BS—blood samples and US—urine samples, collected at different time periods, control—untreated control.

In the fluorescence spectra of the blood serum samples, one could clearly observe the presence of two peaks, at ~474 nm and ~513 nm (Figure 2a). However, the peak observed at ~474 nm was also detected in the blood serum sample of the untreated control (dark green in Figure 2a, i.e., emission due to constituents of isotonic PBS, mostly water), leading to the conclusion that this emission wavelength was not specific for serum SPION-PEG clusters detection. Consequently, the peak observed at ~513 nm remained the only one associated with the presence of SPIONs-PEG clusters in the serum (visible as a large smooth peak in the SPION cluster solution—light blue in Figure 2a—and absent from the untreated blood serum samples—dark green in Figure 2a).

The highest fluorescence was detected in samples collected at 15 min. However, signals were still high 30 min, 60 min, and 180 min after i.p. administration, indicating a fast absorption of SPION-PEG clusters through i.p administration.

#### 3.3.2. Blood Serum—i.v.

Through i.v. administration, SPION-PEG clusters showed high fluorescence at 24 h, but not as high as determined at i.p administration at 24 h.

#### 3.3.3. Urine—i.p.

The fluorescence spectra of urine samples showed an emission peak at ~420 nm, as was evidenced in the spectrum of SPION-PEG clusters; therefore, this peak was associated with the presence of NPs in the urine samples. Due to the short time period in which the rats produced urine, the urine samples were only collected only 3 h (180 min) after i.p. administration (Figure S1b). Therefore, compared to the untreated control, in the spectrum of the urine sample collected 3 h after i.p administration, a visible peak was observed at ~420 nm—corresponding to SPION-PEG clusters. As shown in Figure 2b, the height of the peak at ~420 nm reached a maximum at 12 h post-treatment and started decreasing at 24 h post-administration. This indicates a good blood circulation time and clearance of the SPIONs-PEG clusters.

#### 3.3.4. Urine—i.v.

SPION-PEG clusters administered by i.v. revealed a low concentration in urine at 24 h, which was similar to the concentration detected 24 h after i.p. administration.

### 3.4. Histopathology Studies of Rat Organs

#### 3.4.1. Hematoxylin-Eosin Staining

**Figure 3:**
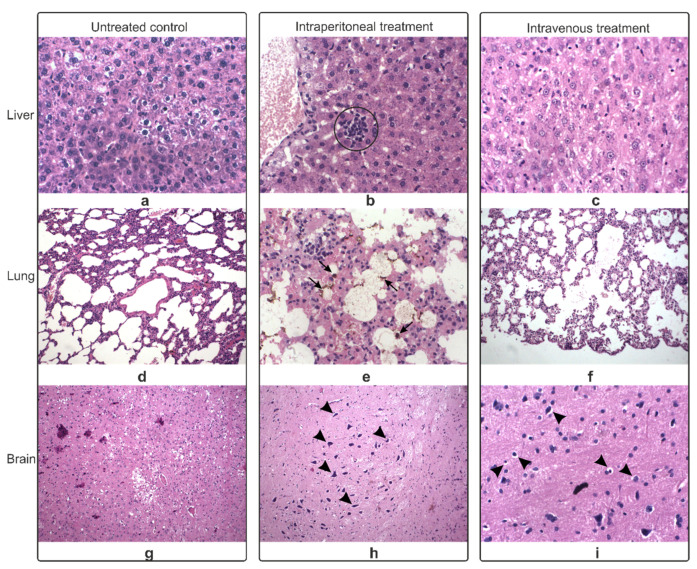
Histological examination of the liver (**a**–**c**), lung (**d**–**f**), and brain (**g**–**i**), harvested 24 h after intraperitoneal and intravenous administration of SPION-PEG clusters and compared to the untreated controls; circle = lymphocyte aggregates, arrows = ferric pigments, arrowheads = shriveled pyramidal neurons (**h**) and hypochromic nuclei (**i**); 40× magnification.

The most relevant pathology-associated modifications were only observed 24 h after i.v. and i.p. administration for SPION-PEG clusters in the lung, liver, and brain, as compared to untreated controls (Figure 3). The spleen, kidney, and skin had no significant alterations (Supplementary Figure S2).

The examined liver showed signs of toxic hepatitis, which could be generated only by the administered SPION-PEG clusters. Both i.p. and i.v. samples had either lymphocyte aggregates or inflammatory infiltrates in the parenchyma (Figure 3a–c). The damages observed in the lungs were consistent with signs of bronchopneumonia with macrophages with ferric pigments and extended areas of emphysema (Figure 3d–f). The brain presented shrunk pyramidal neurons (Figure 3h) and hypochromic nuclei (Figure 3i). Both pathologies are signs of hypoxia, which correlated with the results observed in the lung.

#### 3.4.2. Epoxy Tissue Staining

The organs harvested at 15, 30, 60, and 180 min and those harvested 6 and 12 h after i.p. treatment were within normal parameters. The focus was again on the organs harvested at 24 h post i.p. and i.v. treatment.

**Figure 4:**
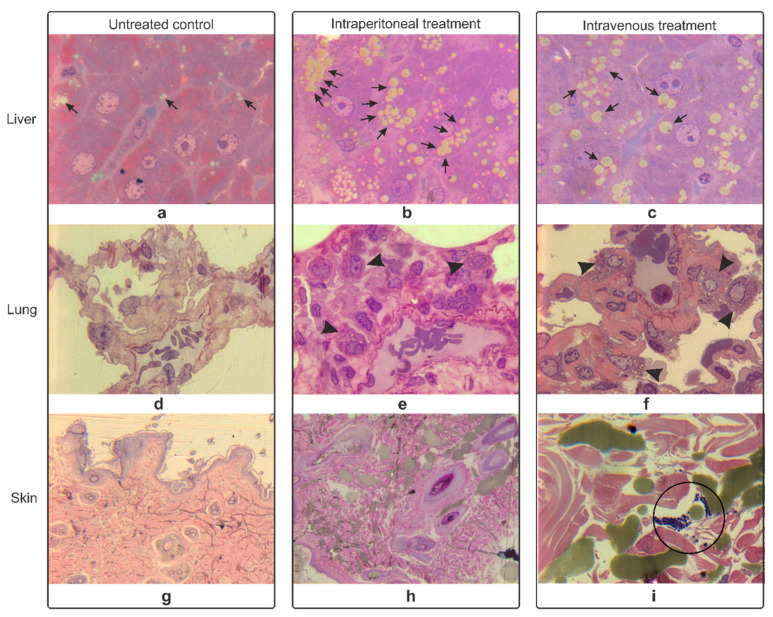
Semi-thin sections of the liver (**a**–**c**), lung (**d**–**f**), and skin (**g**–**i**) examined at 24 h post-intraperitoneal-treatment and 24 h post-intravenous-treatment and compared to the untreated controls; arrows = lipid accumulations, arrowheads = activated macrophages, circle = dense accumulations; 100× magnification.

A closer look at the organs’ histology revealed slightly altered intracellular differences of the liver, lung, and skin (Figure 4), while the spleen, kidney, and brain were within normal parameters (Supplementary Figure S3). A fat liver was observed compared to the untreated control, with a high density of lipid droplets for the i.p. probe (Figure 4b) and large lipid droplets for the i.v.-treated probe (Figure 4c).

The changes observed in the lungs were consistent with the hematoxylin-eosin-stained samples. Sings of bronchopneumonia with erythrocytes trapped in the alveoli along with activated macrophages were observed in the treated samples (Figure 4e,f).

The skin had dense accumulations associated with SPION-PEG clusters in the i.v.-treated probes (Figure 4i).

### 3.5. Electron Microscopy Analyses of Rat Organs

The blood analysis showed that the highest nanoparticle concentration was present at 15 min post-SPION administration (Supplementary Figure S1). The histopathology analysis showed that the spleen, brain, and skin were the least affected organs in terms of pathology-associated modifications at 6, 12, and 24 h; therefore, these organs were left out for the short time evaluation SPION-PEG cluster tracking by TEM. However, the absence of SPION-PEG clusters from these organs does not necessarily indicate that they were completely missing.

#### 3.5.1. Liver

**Figure 5:**
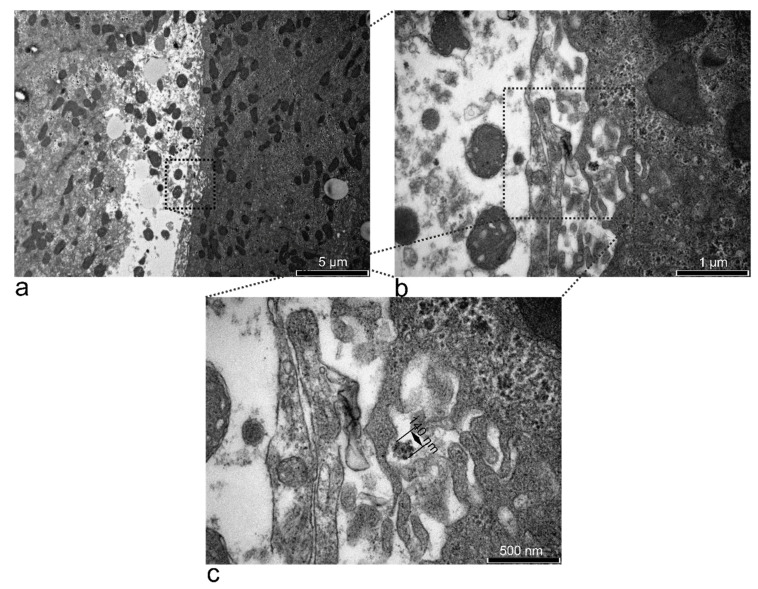
TEM micrographs showing the Disse space (limit between a hepatocyte and a blood vessel) of a rat treated with SPION-PEG clusters for 15 min (**a**). At higher magnification (**b**), microvilli of the hepatocyte seem to have sequestered a small electron-dense cluster that resembles SPIONs-PEG. The cluster was 140 nm in diameter (**c**) and could be a larger nanocluster that maintained its form due to the large fenestrae (~100 nm) of liver sinusoids.

**Figure 6:**
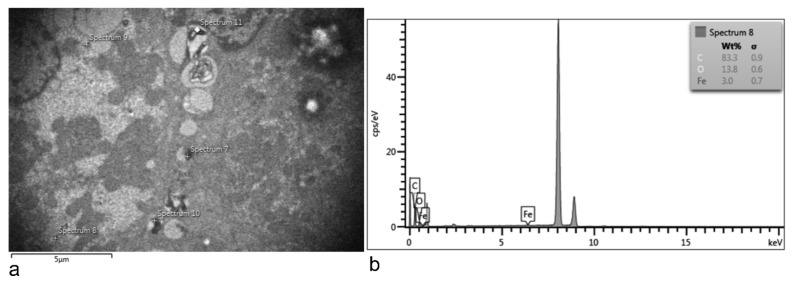
EDX examination of the 12 h post-treated liver. TEM image of the hepatocytes (**a**); EDX spectra of one of the electron-dense accumulation observed inside a hepatocyte. The large unattributed peak represents Cu, detected from the sample grid (**b**).

At 15 min after treatment, the ultrastructure of the liver was not affected, compared to the untreated control. However, clusters resembling SPIONs-PEG were detected in the extracellular space (Figure 5). An EDX examination of the liver revealed Fe deposits in the electron-dense formations (Figure 6).

At 15, 30, 60, and 180 min, and at 6 h, electron-dense accumulations were detected in hepatocytes, indicating a hepato-biliary-fecal clearance. In contrast, at 12 h, the macrophages (Kupfer cells) had traces of SPION-PEG clusters (Supplementary Figure S4), which indicates a clearance thorough the reticulo-endothelial-system [39].

#### 3.5.2. Lung

**Figure 7:**
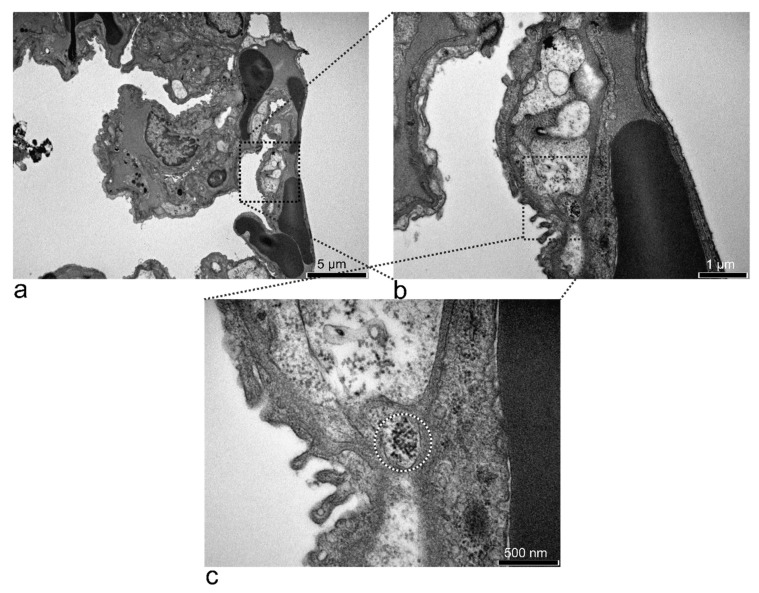
TEM micrographs of the lung treated with SPION-PEG for 15 min (**a**). At a higher magnification, a large vesicle was observed (**b**). The vesicle (black and white circle) had electron-dense particles inside, resembling 10 nm SPIONs (**c**).

Lungs had no pathological and ultrastructural modifications at 15 min after the SPION-PEG cluster i.p. administration. A vesicle with electron-dense accumulations was observed near a blood vessel inside an interalveolar septum (Figure 7). SPION-PEG clusters could not be detected by TEM at any of the other time points in the lung.

#### 3.5.3. Kidney

At 12 h, the urine had the highest accumulation of SPION-PEG clusters, indicating that they had passed through the kidneys in higher concentrations before that time point.

**Figure 8:**
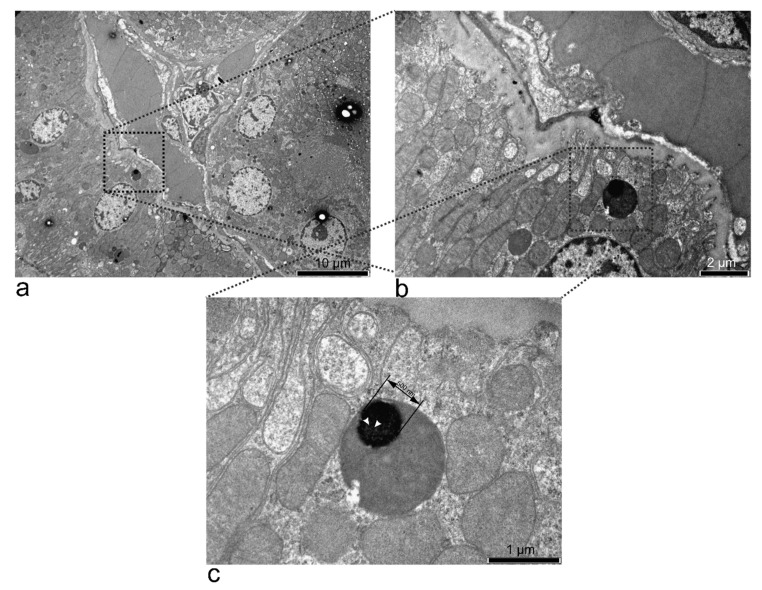
TEM micrographs of the kidney treated with SPION-PEG clusters for 15 min (**a**). At a higher magnification, a dark lysosome was observed (**b**). The lysosome had a large electron-dense formation inside, with visible smaller particles (white arrows) that could be the 10 nm SPIONs (**c**).

According to the blood spectra, 15 min after i.p. treatment, the amount of SPION-PEG clusters reached the highest levels in the blood. This indicated that the nanoparticles had passed through the kidneys’ capillaries; therefore, this organ expected some changes. However, the ultrastructure of the kidney was within normal parameters. The lysosomes in many collector tubes had electron-dense formations close to the shape of SPION-PEG clusters (Figure 8). At 60 min, ~200 nm clusters could be seen trapped in the basement membrane between the podocyte pedicels and the endothelial cells (Supplementary Figure S5). These data indicate that the 50 nm SPION-PEG clusters passed the nephrocyte size barrier either directly through the nephrin network and/or by transcytosis [38].

#### 3.5.4. 24 h after i.p. and i.v. Treatment

**Figure 9:**
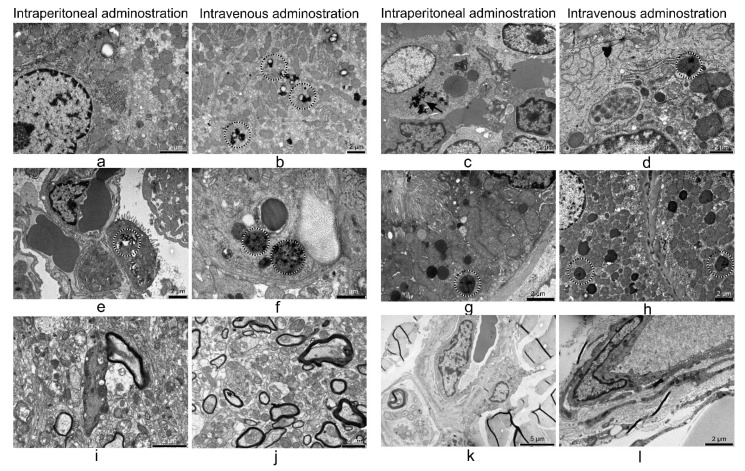
TEM micrographs of the organs examined 24 h after intraperitoneal or intravenous treatment with SPION-PEG clusters; liver (**a**,**b**)**,** spleen (**c**,**d**), lung (**e**,**f**), kidney (**g**,**h**), brain (**i**,**j**), and skin (**k**,**l**). Arrows and circles indicate the presence of electron-dense accumulations inside lysosomes or dispersed in the cytosol.

The ultrastructural examination of the organs harvested 24 h after i.p. and i.v. treatment (Figure 9) showed a high number of electron-dense particles in the macrophages and parenchymal cells of the liver, spleen, lung, and kidney, while the brain and skin were within normal parameters. The untreated controls of these organs can be observed in Supplementary Figure S6.

## 4. Discussion

In this study, we used superparamagnetic magnetite clusters (SPION-PEG clusters), obtained by methods that have proven their superparamagnetic capacities and potential biomedical applications [41,51,52] for in vivo biodistribution and clearance. A summary of the findings can be consulted in Table 1.

A maximum of fluorescence corresponding to SPION-PEG clusters was observed in blood samples 15 min after i.p. treatment and in urine 12 h after i.p. treatment, and this decreased at 24 h, which signifies a plasma half-life of 12 h [53]. This indicates good circulation time and clearance, making SPION-PEG clusters efficient for imaging and/or treatments and non-toxic [54]. This long circulation property of the SPION-PEG clusters is determined by the PEG, which increases both circulation time and biocompatibility [55,56]. The clusters were distributed to several tissues and could be observed by TEM after as little as 15 min in the liver and kidney and by histology staining in the skin and liver at 24 h. The histological and ultrastructural examinations showed that SPION-PEG clusters caused slight morphological alterations in the liver cells. Electron-dense particles were present in the kidney, spleen, and lungs at 24 h, but no ultrastructural alterations were seen in these organs’ cells, where SPION-PEG clusters accumulated. Our SPION formulation reached several organs, both with and without the reticulo-endothelial system (RES), where they were found as unique clusters and not clumped together in aggregates. This aspect indicates good colloidal stability in the blood and increases their ability to distribute throughout the tissue, thus increasing their tissue distribution and MRI visualization window to more than 12 h [55]. As a comparison, the FDA-approved (Food and Drug Administration) iron oxide, called ferumoxytol, has a 14 h circulation half time in humans and 2 h in rodents, and a peak of RES accumulation at 12–24 h [57]. When clusters began to be degraded, this process was slow and gradual from the cluster edge (PEG) to the core, keeping the SPION crystallites at the core intact for a longer time, thus increasing their capacity for imaging and hyperthermia to longer than 24 h post-injection.

Unlike the untreated control, fatty liver was observed with a high density of lipid droplets for the i.p. probe (Figure 4b) and large lipid droplets for the i.v.-treated probe (Figure 4c). This would suggest a certain impairment of the lipid metabolism or inflammation, especially for the i.p.-treated lot. Most likely, the entire SPION-PEG cluster dose passed first through to the liver, before spreading throughout the body [58]. This lipid build-up in the hepatocytes seems to be typical for in vivo nanomaterial treatments [59,60,61]. It is most likely due to reactive oxygen species accumulation, which blocks mitochondrial lipid peroxidation and lipid export [62].

Similarly to our results, other findings have also shown that the iron levels in the blood serum were high at 24 h after administration of SPIONs, with no morphological damages in various organs [63,64]. Jarockyte et al. [65] showed that even after six months post-treatment, SPIONs were still not cleared from the injection site in rats, and this was dependent on the dose of nanoparticles used. Fu et al. [66] showed that iron oxide dextran-coated clusters of 150 nm were biocompatible in vitro and in vivo, and did not determine toxicity in the blood, liver, or kidney for up to 3 months. In their study, the SPION clusters were administered intra-abdominally, and the clusters were distributed predominantly to the lymph nodes and the heart, liver, kidney, spleen, and lungs.

Briley-Saebo et al. [67] showed that SPIONs were dispersed equally between the endothelium and Kupfer cells in rat liver, an aspect that we could also observe. Still, no details about the morpho-anatomical changes in this organ were described in the paper. Also, Deng et al. (2021) [15] tested their biocompatible 4 nm SPIONs, observed no damages in the hepatic tissues, and showed the safe applicability of those NPs as contrast agents. The use of SPIONs-PEG in vivo was reported before [68,69]; however, no other studies are investigating the distribution and histopathological effects of 50 nm SPION-PEG clusters on filtering organs (liver, spleen, kidney) as far as we are aware.

A study showed that SPIONs could induce histopathological and biochemical alterations in the lungs of mice [70]. The described ultrastructural damages induced by NPs in lung phagocytes were size-dependent and targeted mainly the mitochondrial membranes [71]. In our study, we also found macrophages with electron-dense accumulations, suggesting the presence of SPIONs in the lung at 24 h post-administration. We consider that the observed damages, determined by local inflammation or anoxia, may be influenced by the large hydrodynamic dimension or by the cluster aggregation in the low-flow capillaries, as concluded by others [72,73]. However, the observed damages of the liver and lung were temporary, and studies show that inflammation is slightly higher in the first few days and then reduces to normal in the next 48 h to 14 days [37], and also that SPIONs determine a reduction of the inflammation genes’ expression [74,75]. This is true for PEG, polyvinyl alcohol, polydopamine, pluronic, dextran, arginine, or oleic-acid-coated SPIONs [37,55,76].

Our 50 nm SPION-PEG nanoclusters might have reached most organs by entering first the lymphatic system from the abdominal area, as previously suggested [66,77], which later connected to the bloodstream. This aspect is also suggestive of the fact that the clusters reached most organs through the process known as enhanced permeability and retention effect, making them ideal candidates for passive tumor targeting and treatment [53,78,79].

SPION-PEG cluster rapid distribution through the lymphatic system may also explain the fast localization of clusters at the lung alveolar level. This aspect was exploited for MRI, detecting early-stage metastatic lung tumors in mice with SPIONs [80]. Using polyvinyl alcohol microbubbles loaded with SPIONs, Barrefelt, et al. [81] observed that SPIONs were distributed throughout the liver and lungs as little as 10 min after i.v. administration, where they mainly accumulated at the macrophage level. Due to their larger size (550 μm), they induced local anoxia and inflammation and their presence was observed in large clusters at 1 and 6 weeks after administration, when fibrosis was also associated. All these factors considered, they concluded that the SPION microbubbles were suitable for i.v. treatments. Similarly, Cho et al. [82], delivered intratracheally Cy5.5-SPIONs to rats and observed a dose-dependent inflammation of the lungs, and still considered them to be “good candidates for use in pulmonary delivery vehicles and diagnostic probes”. The inflammation present in the lungs can be both a good and a bad outcome of the SPION treatment; therefore, it should be carefully considered. Inflammation can be the trigger of metastasis, but also the trigger of efficient antitumor immunocompetence [57].

Interestingly, our SPION-PEG clusters of 50 nm dimension managed to pass through the renal system as soon as 15 min after administration and get into the urine in significant concentration, without affecting the kidney histology. Their relatively large dimension for the renal filtration system (50 nm) raises questions about the mechanisms of passage through the filtration slits of the Bowman capsule. Smaller than 10 nm SPIONs may be capable of freely traversing the nephrin mesh, and larger ones should be captured by tissue macrophages [83]. A recent study by Balas, et al. [84] showed that iron oxide NPs covered with polymeric micelles (~21 nm) were most likely transformed to their ionic form starting at day 3 post-administration and passed through the nephron, determining antioxidant enzymatic imbalance and increasing expression of kidney injury markers, inflammation, and oxidative stress. All these effects were subdued by day 7 post-administration. Another study, using 20 nm (DLS-measured) SPION clusters covered in 10 kDa PEG obtained similar results to ours—free passage into the urine (despite the large size) and avoidance of RES. They too, detected SPION presence in the kidney as soon as 30 min after administration; the SPIONs were already located in lysosomes of proximal tubules epithelial cells. They explained the clusters’ passage through the narrow filtration slits by referring to clusters’ flexibility to change form to squeeze through the small space. Their conclusion was that these effects were due to the use of high-density PEG [85]. Our study could not detect SPION-PEG clusters in renal macrophages and kidney histology and ultrastructure were unchanged. Still, their presence was clearly detected in the basement membrane, collector tubes, and rat urine, with the same cluster fluorescence signature.

Imam et al. [86] showed that SPIONs could impair the blood–brain barrier (BBB) integrity in vitro and the 10 nm particles used in the in vivo study altered the water retention in the brain [86]. A different study also showed damages induced in the rat brain treated with SPIONs-PEG. Still, the synthesized nanoparticles had under 10 nm diameter and their translocation in the brain was, thus, considered possible [87]. However, our 50 nm SPION-PEG clusters did not seem to penetrate the BBB and could not be located in the brain at neither short- nor long-term treatment.

In our study, SPION-PEG clusters accumulated at the skin level as soon as 24 h after administration, without inducing histopathological changes. Although in vitro and in vivo studies have repeatedly shown no significant or only transient effects on skin cells after i.v. or topical administration of various SPIONs formulation [88,89,90], recent studies have indicated that the observed effects may take place after months or longer. Human skin was shown to naturally contain iron oxides in the form of magnetite in the dermal layer, which may be considered now a form of long-term tissue deposition for iron intake, from either ingested or inhaled sources during lifetime [91]. It is worth mentioning that FDA-approved SPION-based nano-treatments did not obtain approval, again due to many unexpected side effects, which included skin-related alopecia, skin pigmentation, and desquamation of the skin called ‘foot and hand syndrome’ [92,93]. The skin has a large number of resident macrophages and a recently discovered possibility to produce biogenically magnetite [94]; therefore, the skin should be better analyzed for long-term effects of iron oxide administration/intoxication.

Thus, the obtained SPION-PEG clusters are suitable for many in vivo applications, either as enhanced contrast agents or as an alternative treatment of various tumors, through hyperthermia. Studies have shown that clustering of small SPIONs increases contrast due to magnetic field inhomogeneities and offers better MRI resolution than current FDA-approved Ferucarbotran, used at the same dose [28,95]. For future studies, the aspect of cluster size may be further addressed to avoid aggregation, as from the standpoint of histology, ultrastructure, and clearance, we consider the SPION-PEG clusters biocompatible platforms that are appropriate for application after specific adjustments and upgrades. Thus, the combination of small SPIONs (10 nm) embedded in a matrix of high-density PEG (10 kDa) at a controlled size of 50 nm radius offers several enhanced properties that make it better suited for in vivo therapy and/or imaging.

Engineered nanomaterials result from technological ingenuity, and their expansion brought a series of advantages for biomedicine and threats to human health [96]. Conventional cancer therapies employ the use of combined radiotherapy with chemotherapy and surgery, methods that often lead to autoimmune responses and resistance to drugs [17]. The synergistic action of conventional cancer therapies with the targeted treatment of tumors leads to a decreased dose administration and higher success rates [14,97,98]. However, if nanoparticles are used as an alternative treatment, the effects that they could have in surrounding tissues should also be considered. Long exposure to SPIONs or other types of NPs was shown to have effects on future generations in mice, leading to the conclusion that even if SPIONs are used with good intentions to treat a form of cancer, their retention in the organism could lead to unforeseen problems [10].

## 5. Conclusions

This study aimed to show whether nanometer SPION-PEG clusters are prone to accumulate in certain organs and to induce histological and ultrastructural changes after intraperitoneal or intravenous injections. Electron-dense accumulations consistent with SPION-PEG dimensions were detected in organs such as the liver, spleen, lung, kidney, and skin, indicating that the biocompatible NPs entered the system successfully and were eliminated, as shown by the spectrophotometric analysis of the urine. This investigation provides a solid basis for further exploiting the obtained SPION-PEG clusters as contrast agents for tumor inhibition through hyperthermia.

## Figures and Tables

**Table 1:** Summary of SPION-PEG cluster distribution and effects observed in our study.

Organ/Tissue SPION Concentration/Effects	i.p.							i.v.
	15 min	30 min	1 h	3 h	6 h	12 h	24 h	24 h
blood	high	high	high	high	high	medium	high	high
urine	-	-	-	low	low	high	low	low
liver	u.u. SPION ex	n.p.m.	n.p.m.	n.p.m.	n.p.m.	n.p.m.	inflammatory infiltrates, high density of small lipid droplets, high number of electron-dense particles in the macrophages and parenchymal cells	inflammatory infiltrates, a few large lipid droplets, high number of electron-dense particles in the macrophages and parenchymal cells
spleen	n.p.m.	n.p.m.	n.p.m.	n.p.m.	n.p.m.	n.p.m.	n.p.m. high number of electron-dense particles in the macrophages and parenchymal cells	n.p.m. high number of electron-dense particles in the macrophages and parenchymal cells
lung	u.u. SPION in	n.p.m.	n.p.m.	n.p.m.	n.p.m.	n.p.m.	SPION-loaded and activated macrophages, extended areas of emphysema, local hypoxia, high number of electron-dense particles in the macrophages and parenchymal cells	SPION-loaded and activated macrophages, extended areas of emphysema, local hypoxia, high number of electron-dense particles in the macrophages and parenchymal cells
kidney	u.u.	n.p.m.	u.u. SPION in	n.p.m.	n.p.m.	n.p.m.	n.p.m. high number of electron-dense particles in the macrophages and parenchymal cells	n.p.m. high number of electron-dense particles in the macrophages and parenchymal cells
brain	n.p.m.	n.p.m.	n.p.m.	n.p.m.	n.p.m.	n.p.m.	shriveled pyramidal neurons, hypochromic nuclei, local hypoxia	shriveled pyramidal neurons, hypochromic nuclei, local hypoxia
skin	n.p.m.	n.p.m.	n.p.m.	n.p.m.	n.p.m.	n.p.m.	n.p.m.	d.a.

i.p = intraperitoneal, i.v. = intravenous, d.a. = dense accumulations, n.p.m. = no pathological modifications, u.u. = unaffected ultrastructure, SPION ex = SPION clusters in the extracellular space, SPION in = SPION clusters in the intra-alveolar septum.

## Data Availability

All data are available from the corresponding author, upon reasonable request.

## Supplementary Materials

The following are available online at https://www.mdpi.com/article/10.3390/nano11092184/s1. Figure S1: The fluorescence spectra of blood (a) and urine (b) samples examined at 15, 30, 60, and 180 min post intraperitoneal administration of SPIONs. BS 15-180 = blood samples, US = urine samples examined at different time periods, SPION = superparamagnetic iron oxide nanoparticles. The most prominent peaks were detected at 15 and 30 min for blood samples and decreased for 60 and 180 min. Compared to the control, the urine sample responded at the specific wavelength for SPIONs; Figure S2: Histological examination of the spleen (a–c), kidney (d–f), and skin (g–i) harvested at 24 h post intraperitoneal and intravenous administration of SPIONs and compared to the untreated controls; the organs were within normal parameters; 40× magnification; Figure S3: Semithin sections of the spleen (a–c), kidney (d–f), and brain (g–i) examined at 24 h post intraperitoneal treatment and 24 h post intravenous treatment and compared to the untreated controls; the organs were within normal parameters; 100× magnification; Figure S4: TEM micrographs of the liver treated at 30 min (a), 60 min (b), 180 min (c), 6 h (d), and 12 h (e–f). Black arrows indicate SPIONs-PEG clusters present in hepatocytes (a–d) and in the macrophages (12 h); Figure S5: TEM micrographs of the kidney harvested at 60 min post intraperitoneal injection of SPIONs-PEG clusters (a), with a close-up of a cluster trapped in the basement membrane (b); Figure S6: TEM micrographs of the untreated organs; liver (a), spleen (b), lung (c), kidney (d), brain (e), and skin (f).
