# Supplementary material for: In Vivo Distribution of Poly(ethylene glycol) Functionalized Iron Oxide Nanoclusters: An Ultrastructural Study
Source: Nanomaterials (Basel). 2021 Aug 25;11(9):2184. doi: 10.3390/nano11092184 (PMC8469409; doi:10.3390/nano11092184)
Supplement: Supplementary file 1 [file nanomaterials-11-02184-s001.zip › nanomaterials-1342080-supplementary.pdf]

Supplementary material

In Vivo Distribution of Poly(ethylene glycol) Functionalized Iron Oxide Nanoclusters: An Ultrastructural Study

Maria Suciu [1,2], Claudiu Mirescu [1], Izabell Crăciunescu [3], Sergiu Gabriel Macavei [3], Cristian Leoștean [3], Răzvan Ştefan [4], Loredana E. Olar [4], Septimiu-Cassian Tripon [1,2], Alexandra Ciorîță [1,2,*] and Lucian Barbu-Tudoran [1,2,*]

[1] Electron Microscopy Centre, Faculty of Biology and Geology, Babeș-Bolyai University, 44 Republicii St., 400015 Cluj-Napoca, Romania; suciu.maria@ubbcluj.ro (M.S.); claudiu.mirescu@gmail.com (C.M.); septimiu.tripon@ubbcluj.ro (S.-C.T.)

[2] Integrated Electron Microscopy Laboratory, National Institute for Research and Development of Isotopic and Molecular Technologies, 67-103 Donat St., 400293 Cluj-Napoca, Romania

[3] Physics of Nanostructured Systems Department, National Institute for Research and Development of Isotopic and Molecular Technologies, 67-103 Donat, 400293 Cluj-Napoca, Romania; izabell.craciunescu@itim-cj.ro (I.C.); sergiu.macavei@itim-cj.ro (S.G.M.); cristian.leostean@itim-cj.ro (C.L.)

[4] Research Centre for Biophysics, Life Sciences Institute, Faculty of Veterinary Medicine, University of Agricultural Sciences and Veterinary Medicine Cluj-Napoca, , 3-5 Manastur St., 400372 Cluj-Napoca, Romania; rstefan@usamvcluj.ro (R.Ş.); loredana.olar@usamvcluj.ro (L.E.O.)

* Correspondence: alexandra.ciorita@ubbcluj.ro (A.C.); lucian.barbu@ubbcluj.ro (L.B.-T.)

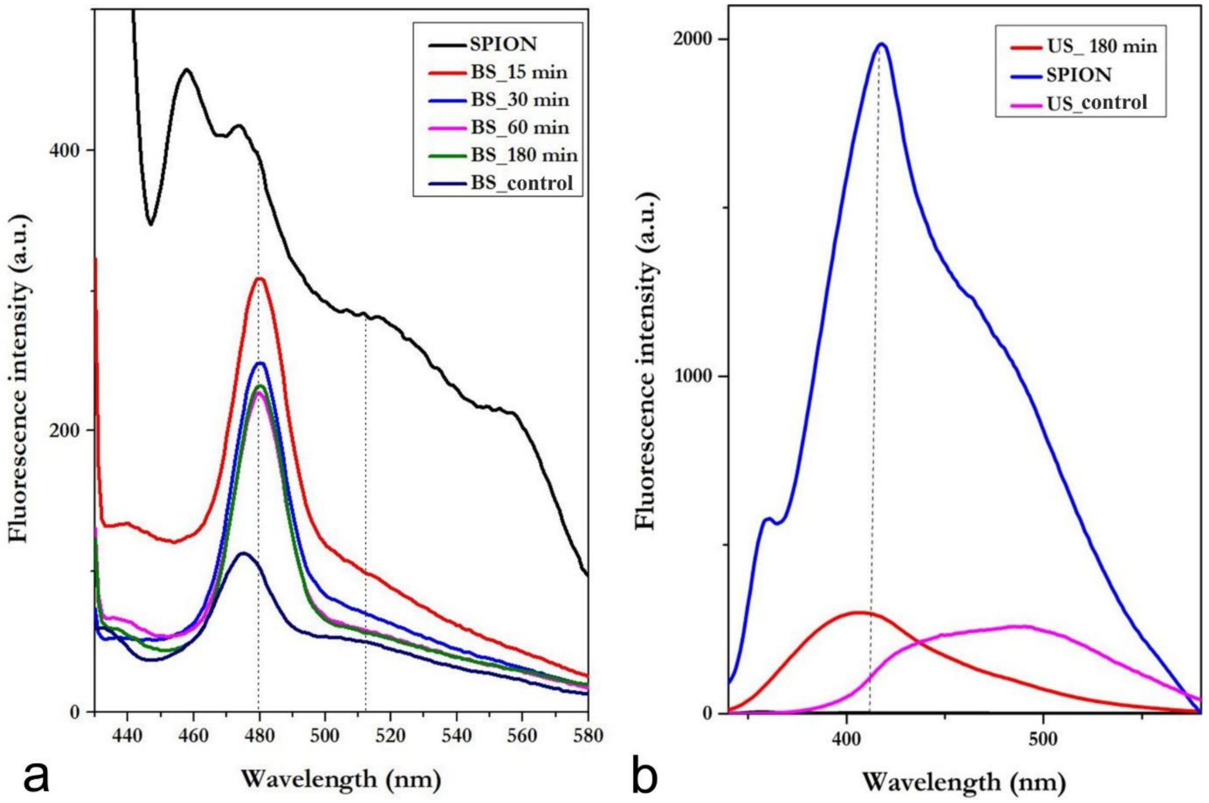

Figure S1. The fluorescence spectra of blood **(a)** and urine **(b)** samples examined at 15, 30, 60, and 180 min post intraperitoneal administration of SPIONs. BS 15-180 = blood samples, US = urine samples examined at different time periods, SPION = superparamagnetic iron oxide nanoparticles. The most prominent peaks were detected at 15 and 30 minutes for blood samples and decreased for 60 and 180 minutes. Compared to the control, the urine sample responded at the specific wavelength for SPIONs.

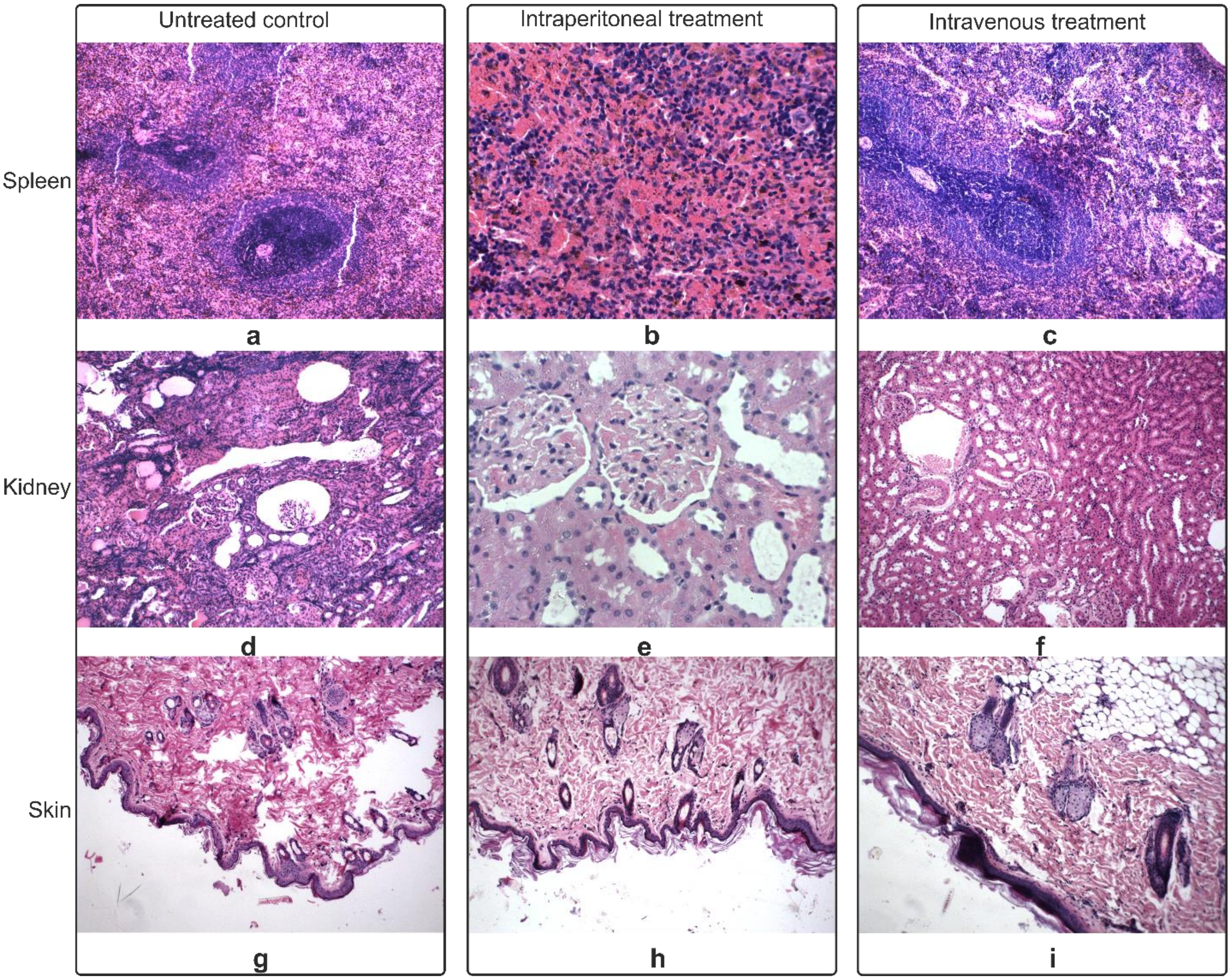

Figure S2. Histological examination of the spleen **(a-c)**, kidney **(d-f)**, and skin **(g-i)** harvested at 24 h post intraperitoneal and intravenous administration of SPIONs and compared to the untreated controls; the organs were within normal parameters; 40× magnification.

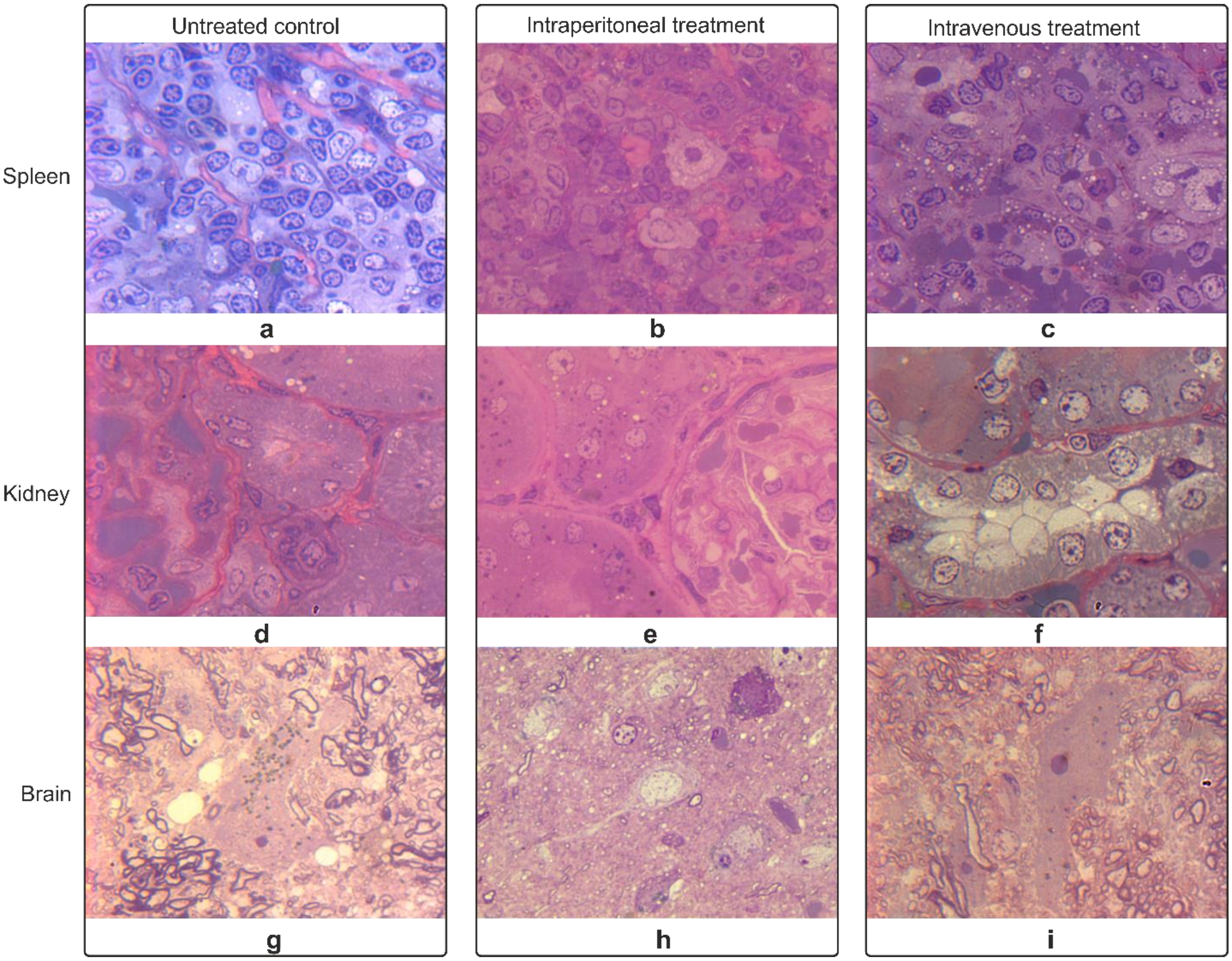

Figure S3. Semithin sections of the spleen **(a-c)**, kidney **(d-f)**, and brain **(g-i)** examined at 24 h post intraperitoneal treatment and 24 h post intravenous treatment and compared to the untreated controls; the organs were within normal parameters; 100× magnification.

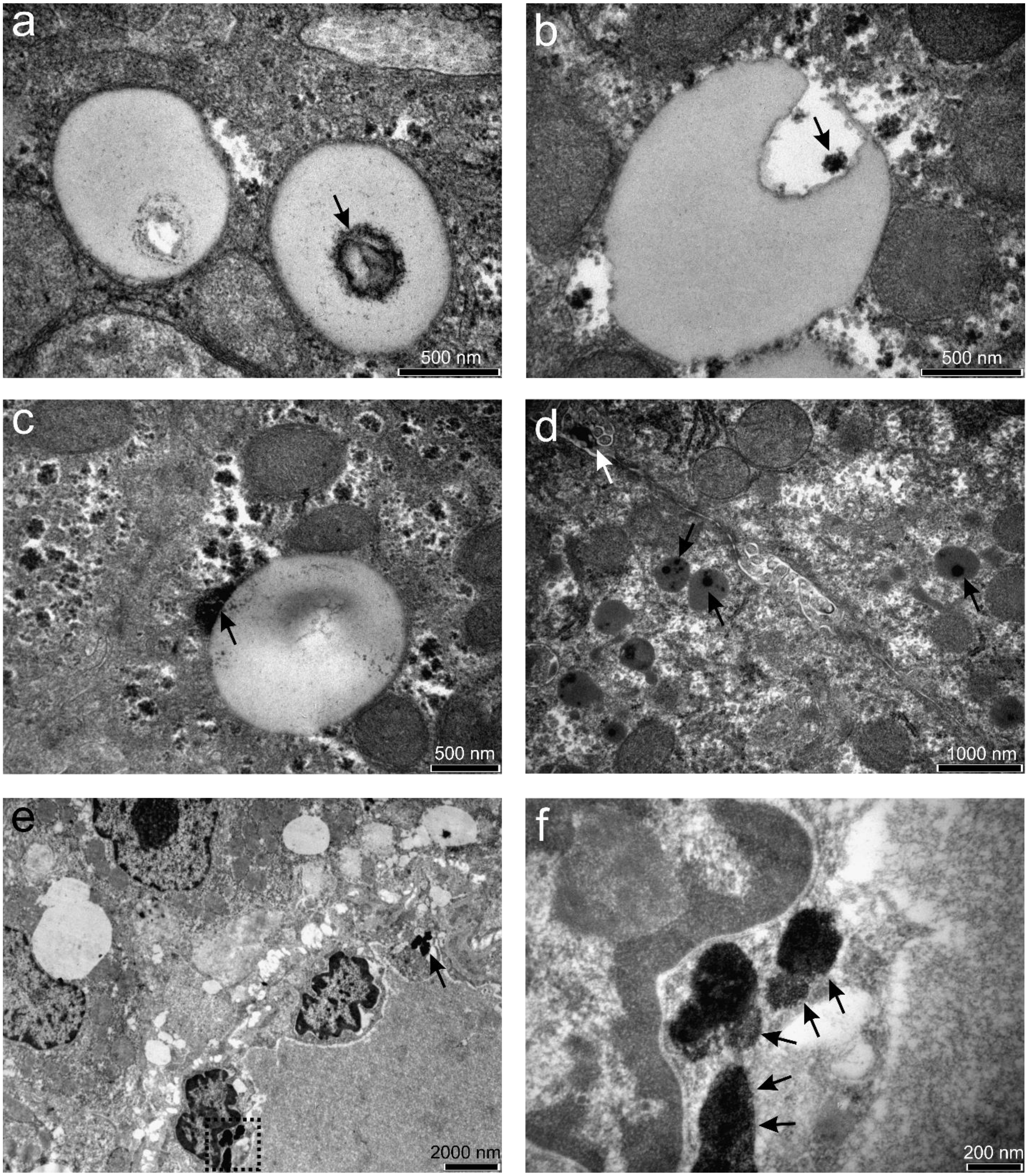

Figure S4. TEM micrographs of the liver treated at 30 min **(a)**, 60 min **(b)**, 180 min **(c)**, 6 h **(d)**, and 12 h **(e-f)**. Black arrows indicate SPIONs-PEG clusters present in hepatocytes **(a-d)** and in the macrophages (12 h).

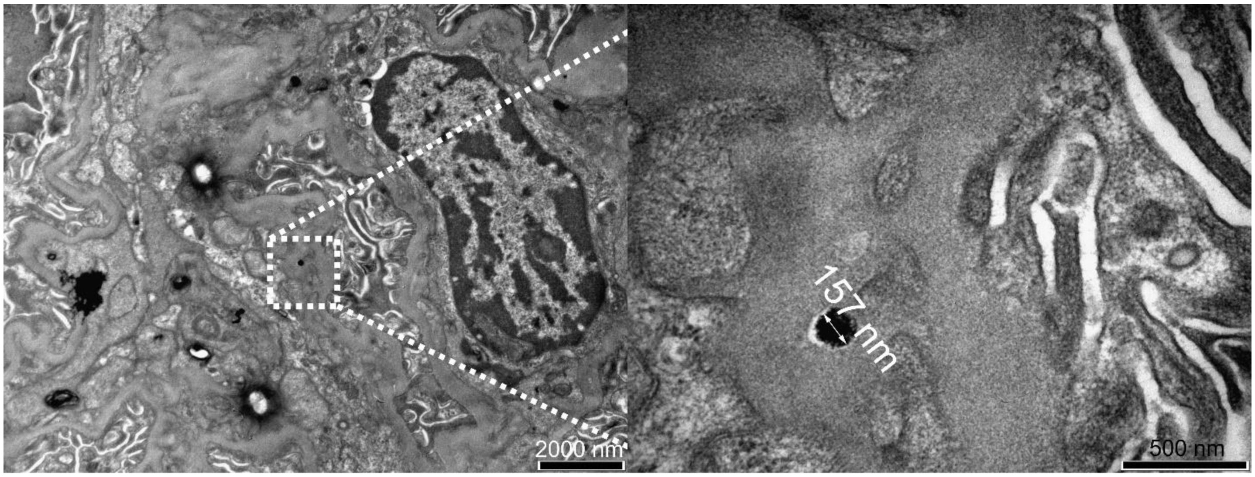

a b

Figure S5. TEM micrographs of the kidney harvested at 60 min post intraperitoneal injection of SPIONs-PEG clusters **(a)**, with a close-up of a cluster trapped in the basement membrane **(b)**.

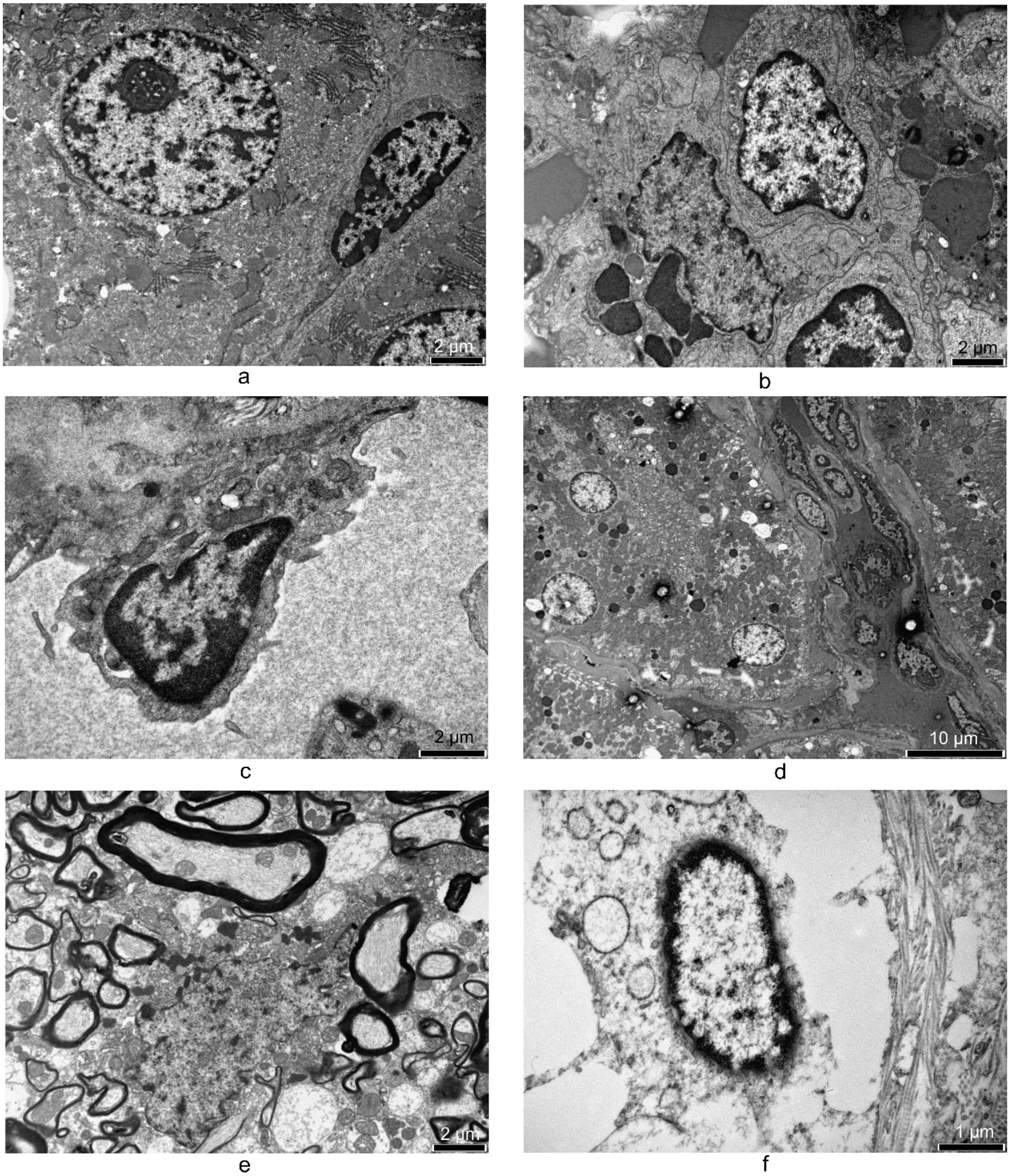

Figure S6. TEM micrographs of the untreated organs; liver **(a)**, spleen **(b)**, lung **(c)**, kidney **(d)**, brain **(e)**, and skin **(f).**
